# The cavity-nest ant *Temnothorax crassispinus* prefers larger nests

**DOI:** 10.1007/s00040-014-0372-4

**Published:** 2014-10-09

**Authors:** S. Mitrus

**Affiliations:** Laboratory of Evolution and Animal Ecology, Department of Biosystematics, Opole University, Oleska 22, 45-052 Opole, Poland

**Keywords:** Sex allocation ratio, Nest choice, Colony size

## Abstract

Colonies of the ant *Temnothorax crassispinus* inhabit mostly cavities in wood and hollow acorns. Typically in the field, nest sites that can be used by the ant are a limited resource. In a field experiment, it was investigated whether the ants prefer a specific size of nest, when different ones are available. In July 2011, a total of 160 artificial nests were placed in a beech-pine forest. Four artificial nests (pieces of wood with volume cavities, ca 415, 605, 730, and 980 mm^3^, respectively) were located on each square meter of the experimental plot. One year later, shortly before the emergence of new sexuals, the nests were collected. In July 2012, colonies inhabited more frequently bigger nests. Among queenright colonies, the ones which inhabited bigger nests had more workers. However, there was no relationship between volume of nest and number of workers for queenless colonies. Queenright colonies from bigger nests produced more sexual individuals, but there was no correlation between number of workers and sex allocation ratio, or between volume of nest and sex allocation ratio. In a laboratory experiment where ant colonies were kept in 470 and 860 mm^3^ nests, larger colonies allocated more energy to produce sexual individuals. The results of this study show the selectivity of *T. crassispinus* ants regarding the size of nest cavity, and that the nest volume has an impact on life history parameters.

## Introduction

The majority of social insects live in nests (Pratt, 2010). The location and other parameters of a nest can affect predation, parasitism, access to food, reproduction, and competition with other colonies (cf. Blüthgen and Feldhaar, 2010), thus finding a suitable nest site is therefore essential for the survival and reproduction of ants (e.g. Foitzik et al., 2004; Blüthgen and Feldhaar, 2010). The availability of nest sites suitable for ants is mostly limited (e.g. Foitzik and Heinze, 1998; Philpott, 2005), and those that are available display major diversity. One of the main nest parameters is the size. Numerous ant species nest in the ground and may extend the nest according to their needs; however, many species find nest sites and use them as found or make only minor alterations (Blüthgen and Feldhaar, 2010).

Ants of the genus *Temnothorax* form small colonies (composed of a dozen, or several dozen up to several hundred individuals) that inhabit small cavities. Depending on the species, they choose for their nests spaces located, e.g. in fallen twigs, under rocks, and in the ground (Seifert, 2007; Czechowski et al., 2012). *Temnothorax crassispinus* ant colonies mostly reside in wood cavities (fallen twigs) and acorns, and less often in the ground or in beech nuts, etc. (Białas et al., 2011; Czechowski et al., 2012). The number of such nest sites is usually limited, and their availability changes seasonally. For this reason, colonies may be forced to move nest locations frequently, up to several times per season, due to the destruction of their previous nest (e.g. when a nest is accidentally crushed or no longer inhabitable as a result of decaying processes) (Herbers, 1989; Herbers and Johnson, 2007).

In laboratory conditions, the choice of nest sites was investigated in *Temnothorax* ants considering many aspects (e.g. Pratt and Pierce, 2001; Dornhaus et al., 2004; Franks et al., 2006, 2008; Robinson et al., 2009; Doran et al., 2013; Kramer et al., 2013; Sasaki and Pratt, 2013). For example, it has been found that ants can select a nest from various sites available, guided by specific attributes, such as entrance size (i.e. they prefer narrow entrances, which are easier to defend) and the amount of light that reaches the cavity (i.e. they prefer shaded nests, choosing nests covered with red foil rather than those covered solely with glass in laboratory studies). During experimental studies, *T. albipennis* ant colonies chose superior nests, even if these were located at a greater distance than nearby nest sites of lower quality (Franks et al., 2008). In natural conditions, a correlation has been shown in the ant *T. nylanderi* (a sibling species of *T. crassispinus*) between the volume of the nest site where the cavity used by an ant colony was situated and the number of workers in a colony (Foitzik and Heinze, 1998). Only estimated volumes of nest site were applied in the mentioned study, as the exact volume of the cavity occupied by ants is usually difficult to measure (branch cavities occupied by *Temnothorax* ants are typically irregularly shaped, amply forked, etc.). The aim of this paper was to investigate whether *T. crassispinus* ants show a preference with regard to the nest cavity size in natural conditions, when having nests of different cavity volume at their disposal. The influence of nest cavity size on life history parameters was also analyzed.

## Materials and methods

### Study organisms

The cavity-dwelling ant *Temnothorax crassispinus* is present throughout Western and Central Europe (Seifert, 2007; Czechowski et al., 2012) and it is a species widely distributed in Poland, known mostly from sites in coniferous forests (Czechowski et al., 2012). They are among the most widely distributed and most common, but also most frequently overlooked ants in Central European habitats: colonies of the species are small, ranging from a few dozen to several hundreds of workers (Seifert, 2007; Czechowski et al., 2012). Colonies of the ant *Temnothorax* are usually monogynous (have one queen) (Heinze and Buschinger, 1988; Seifert, 2007; Czechowski et al., 2012).

### Combined field and laboratory experiment

The fieldwork part of the experiment was conducted on the edge of a beech-pine forest (ca. 5 m from the forest line), in the area where a high density of *T. crassipinus* ant colonies was discovered during previous research (cf. Białas et al., 2011). In July 2011, 160 artificial nest chambers made of wood were placed in two 4 m × 5 m plots located 12 m apart. Such nest chambers fashioned in wood pieces are willingly accepted by *Temnothorax* ants (cf. Foitzik et al., 2003). Nests used in this study were made of pine dowels with a diameter of 15 mm. Each dowel was drilled lengthwise to form a 4 mm hole, which was tightly closed on one side with a beech plug, and partially closed by a splinter of such a plug on the other. Considering that pinewood dowel fragments of different length were used (45, 60, 70, and 90 mm), after calculating the reduction in the length of drilled cavity due to beech plugs inserted in the hole, the final cavity volumes were ca. 415, 605, 730, and 980 mm^3^, respectively. There are no data on volume size of nests used by *T. crassispinus*. However, the ones used in the research correspond to cavity volumes of most frequently naturally occupied acorns by cavity-nest ant *T. curvispinosus* (cf. Pratt and Pierce, 2001), and are similar to cavity volumes of nests used by *T. crassispinus* (pers. obs.). Four nests varying in size were placed in each square meter of experimental plots. Nests were not fixed to the ground, and—just as in natural nesting sites—could therefore be moved, e.g. accidentally dislocated by animals, although this factor added to the difficulty in finding the nests after 1 year.

One year later (10 and 11 July 2012), experimental plots and adjacent areas (a 1 m perimeter around the areas laid out with nests) were thoroughly surveyed by two persons. Artificial nest cavities retrieved and natural nests with ant colonies were transported to a laboratory, where they were carefully opened, the ants captured with an aspirator and counted. In the case of nest chambers containing only workers (i.e. where no eggs or pupae were found), these colonies were classified as “nests containing only a few individuals” (18 artificial nests where 1–4 workers were found, and one nest with a queen and three workers) and were not included in the subsequent experiment.

**Fig. 1:**
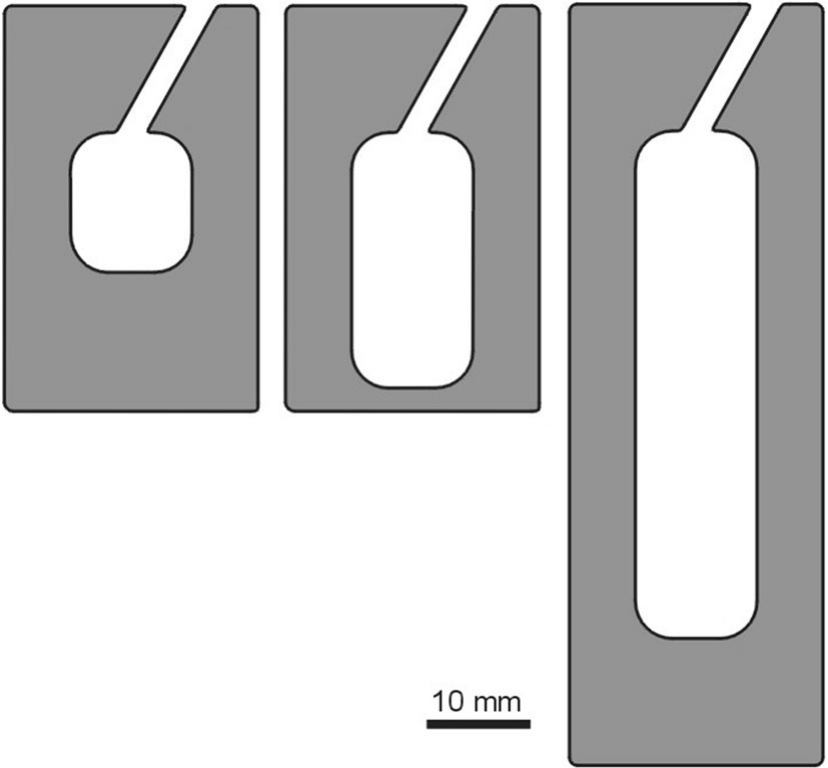
Shape of the plexiglass frames used in laboratory to make artificial nest chambers for colonies of ant *Temnothorax crassispinus*; the nest chambers were of different lengths, and hence in volume. The thickness of the frames was 3 mm

Colonies were transferred to square Petri dishes (10.2 cm × 10.2 cm × 1.9 cm) with a thin plaster base and an artificial nest chamber placed on top. Each nest chamber used in the laboratory was made of a 3 mm thick, fittingly carved plexiglass frame (Fig. 1), sandwiched between two microscope slides or two ½ microscope slides. A piece of cardboard providing the base for the nest was placed between the bottom microscope slide and the plexiglass form. The whole nest was coated with a piece of a red translucent filter from above. All nest chambers used had the same entrances and were of the same shape, differing only in cavity length, and hence in volume; the nests with plexiglass frames fitted between microscope slides were ca. 1,760 mm^3^ in volume, whereas those with plexiglass frames fitted between ½ microscope slides included smaller ones of ca. 470 mm^3^ and larger ones of ca. 860 mm^3^. Ants from the nests made of 45 mm long dowels were transferred to Petri dishes containing 470 mm^3^ artificial chambers, whilst those from the nests made of 60 and 70 mm long dowels to 860 mm^3^ chambers, and those from 90 mm long dowels—to 1,760 mm^3^ chambers. Each colony was placed in a separate Petri dish. The dishes were kept in a Pol-Eko ST 1 thermostatic cabinet maintaining a daily cycle: 14 h of “day” in a temperature of 27 °C and 10 h of “night” in 17 °C. Ants were fed twice a week with frozen fruit flies *Drosophila hydei* and honey.

On 10–11 July while capturing with an aspirator and counting the workers from nests collected on the field, I did not note the presence of any gynes or males in the nests. However, I found the first sexual individuals in the nests (both males and young queens) as early as during the feeding phase on 14 July. Throughout the following weeks, since 24 July, each time I found sexual individuals outside the nest chamber during feeding; I captured and removed them from the Petri dishes. On 6 August, all nests were ultimately opened, and workers and sexual individuals counted (sexual individuals were still present in several nests at that time).

### Laboratory experiment

In order to investigate whether the size of a nest chamber affects sex allocation, I carried out a laboratory experiment in 2013. On 18 June 2013, I collected nests with ants in an area adjacent to that where a field experiment had been carried out from 2011 to 2012 (see above). The date of colony collection depended on the atmospheric conditions; namely it was conducted after a long period of cold and wet weather. Nests were transported to a laboratory, where they were carefully opened, the ants carefully captured with an aspirator and counted. I collected 89 nests containing ant colonies (= nest cavities containing workers, eggs and pupae), including 56 nests with one queen (containing 7–122 workers, 48.0 on average), two nests with two queens (containing 48 and 59 workers), and 31 queenless nests (containing 7–63 workers, 28.8 on average). I also found 12 nesting sites where only workers occurred without eggs and pupae (2–16 workers, 6.8 on average) and one nest with a lone queen.

I used 50 colonies with one queen in the experiment. They were randomly divided into two groups and transferred to Petri dishes. Each dish contained a nest (made as described above) of 470 or 860 mm^3^ volume. These colonies were maintained according to the above-described protocol, except that the day/night regime was 12 h/12 h at temperature 20 °C/10 °C, respectively, until 24 June, and from that day onwards it was gradually shifted to a 14 h/10 h regime at 27 °C/17 °C (the same as artificial spring and summer conditions previously used in experiments on *T. nylanderi* (e.g. Foitzik et al., 2003); the conditions are in accordance with the field conditions at the seasons of the year in central Europe). I observed the first sexual individuals on 15 July. I counted the final number of workers in each colony and the number of sexual individuals produced by each colony on 29 July. Workers and larvae from two colonies (amounting to 151 and 191 workers) placed in Petri dishes with 470 mm^3^ nests at the beginning of the experiment, could not fit in these nests and were not included in further analyses.

### Statistical analysis

In order to estimate the cost incurred of producing sexual individuals, I adopted literature data for the ant *T. nylanderi*, according to which the mass and hence the cost of a young queen being produced, is 3.02-fold higher than that of a male (Foitzik and Heinze, 2000; Foitzik et al., 2003).

In the laboratory experiment, the number of workers at the beginning and their number at the end of the experiment were strongly correlated (*r* = 0.80, *n* = 23, *P* < 0.001 and *r* = 0.76, *n* = 25, *P* < 0.001, for 470 and 860 mm^3^ nests, respectively). The results presented in the analysis of relationship between the number of workers and the allocation of energy in sexual individuals were based on the initial numbers of workers, however, the results obtained using the numbers of workers at the end of the experiment are analogous. Statistical analyses were carried out using the software package Statistica, ver. 10 (StatSoft Inc., 2011). All probability values shown are two-tailed.

## Results

### Combined field and laboratory experiment

**Fig. 2:**
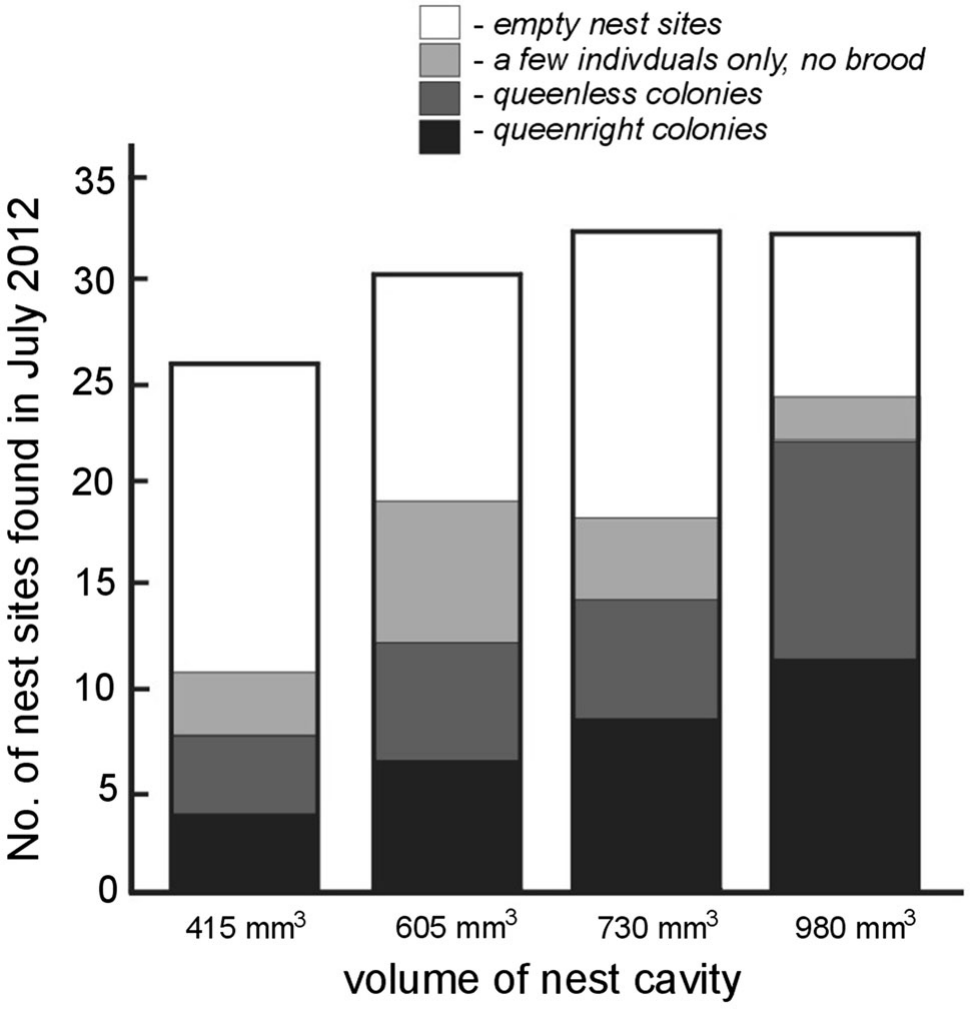
Number of nests of each volume sought out, in which *Temnothorax crassispinus* ant colonies (queenless and queenright), a few individuals without brood, or no ants were found. As part of this field experiment, four nests (one of each volume) per m^2^ were dispersed in July 2011 and collected 1 year later

Out of 160 artificial nests made of pine dowels that had been distributed over the field the previous year, a total of 120 were found (Fig. 2). Colonies of the ant *T. crassispinus* were discovered in 54 nests: 28 of them were queenless colonies (containing 5–44 workers, median: 16.5), 25 colonies had one queen (4–62 workers, median: 27.0), and one colony had three queens and 25 workers. There were more workers in queenright nests than in queenless ones (*U* Mann–Whitney test, *U* = 238.5, *n*_queenright_ = 26, *n*_queenless_ = 28, *P* = 0.030). Additionally, 21 *T. crassispinus* ant colonies were found in natural nesting sites in the experimental plots: 20 in small twigs and one in an acorn that contained an average of 42.1 workers (range 9–119, *n* = 14) and 35.4 workers (14–57, *n* = 7) respectively for queenright and queenless colonies. I did not find any other ant species in the collected artificial nests laid out on the experimental field.

**Fig. 3:**
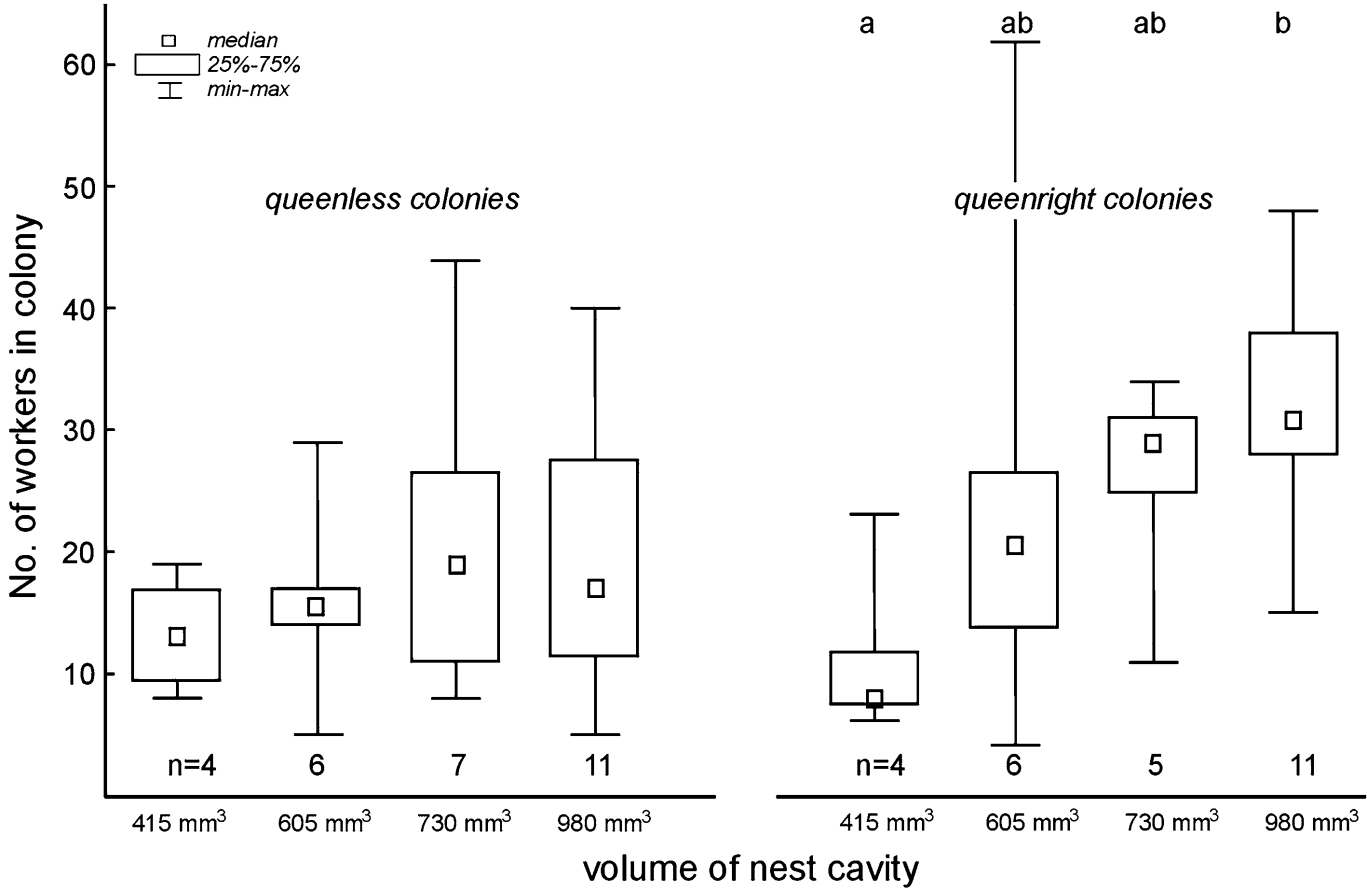
Number of *T. crassispinus* ant workers found in nests of a given volume. Values beyond ranges—number of colonies analyzed. No differences were found in the number of workers between nests for each volume in queenless colonies. For data concerning queenright colonies—different letters indicate the statistical significance after a post hoc test: multiple comparisons of mean ranks for all groups

In July 2012, *T. crassispinus* ant colonies were more frequently found in the nests of larger volume (Fig. 2) (*χ* ^2^ = 10.45, *df* = 3, *P* = 0.015; the analysis concerned the number of nests inhabited by ant colonies—both queenright and queenless—in comparison with nest chambers where ant colonies were not found; namely: both empty nest sites and those with only a few individuals, without brood). Queenright colonies dwelled in nests of different size with the same frequency as queenless colonies (*χ* ^2^ = 0.60, *df* = 3 *P* = 0.97; Fig. 2). In the colonies where queens were present, the number of workers was larger in the nests of larger volume (Kruskal–Wallis test *H*_*df*=3,*n*=26_ = 9.87, *P* = 0.020); I did not note such a relationship for queenless colonies (*H*_*df*=3,*n*=28_ = 1.11, *P* = 0.78) (Fig. 3).

Queenright colonies inhabiting larger nest chambers (and at the same time containing more workers) produced more sexual individuals (*r*_s_ = 0.58, *n* = 26, *P* = 0.0018) and the overall investment of such colonies in sexuals was greater (*r*_s_ = 0.57, *n* = 26, *P* = 0.0025). I did not find any relationship between the chamber size and the proportion of males among sexual individuals (*r*_s_ = 0.11, *n* = 16, *P* = 0.67, analysis was conducted for nests where five or more sexual individuals were obtained).

As regards the queenless nests, I did not observe a relationship between cavity size and the number of sexuals produced (*r*_s_ = 0.00, *n* = 28, *P* = 0.99) or with the sex allocation ratio (*r*_s_ = 0.12, *n* = 28, *P* = 0.53). Neither did I notice a relationship between the cavity size and the proportion of males among sexual individuals (*r*_s_ = −0.42, *n* = 20, *P* = 0.066, analysis conducted for nests where five or more sexual individuals were found).

### Laboratory experiment

The rate of increase in the number of workers (number of workers at the end of experiment/initial number of workers) was negatively correlated to the initial number of workers in the nest in the case of 860 mm^3^ nest chambers (*r*_s_ = –0.78, *n* = 25, *P* < 0.0001), while no such correlation was shown for the colonies from 470 mm^3^ chambers (*r*_s_ = −0.31, *n* = 25, *P* = 0.14). In both cases, this rate was negatively correlated with the investment in sexuals (*r*_s_ = −0.46, *n* = 23, *P* = 0.0027 and *r*_s_ = 0.57, *n* = 25, *P* = 0.0032 for 470 and 860 mm^3^ nest chambers, respectively).

**Fig. 4:**
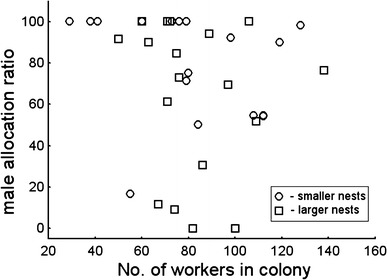
Male allocation ratio in the ant *T. crassispinus* in relation to the number of workers in colonies kept during the laboratory experiment in smaller volume nests (470 mm^3^, *P* = 0.035) and larger volume nests (860 mm^3^, *P* = 0.42) where three or more sex individuals were produced

Larger colonies allocated more energy to the production of sexual individuals (*r*_s_ = 0.67, *n* = 23, *P* < 0.001 and *r*_s_ = 0.75, *n* = 25, *P* < 0.0001, for 470 and 860 mm^3^ nest chambers, respectively, production per colony). However, production per capita was correlated with number of workers for colonies from 860 mm^3^ chambers only (*r*_s_ = 0.40, *n* = 23, *P* = 0.061 and *r*_s_ = 0.70, *n* = 25, *P* < 0.0001, for 470 and 860 mm^3^ nest chambers respectively). There was no relationship between number of workers and sex allocation ratio, taking into consideration the Bonferroni correction (*r*_s_ = −0.51, *n* = 17, *P* = 0.035 and *r*_s_ = −0.21, *n* = 17, *P* = 0.42, for 470 and 860 mm^3^ nest chambers, respectively) (Fig. 4). I did not find a relationship between the per capita investment in sexuals and the proportion of males (*r*_s_ = 0.040, *n* = 17, *P* = 0.88, *r*_s_ = –0.22, *n* = 17, *P* = 0.40, for 470 and 860 mm^3^ nest chambers respectively, analyses made for nests where five or more sexual individuals were produced).

## Discussion

In July 2012, at the end of the field experiment, the *T. crassispinus* ant colonies were more frequently found in larger nest chambers, although equal numbers of nests of each size had been laid out a year before (Fig. 2). I discovered the occurrence of a few workers without brood in numerous artificial nests of all sizes (see Fig. 2). This indicates that these nesting sites were found and penetrated by workers but the chambers of larger volume were more often chosen by ant colonies of the studied species for their nesting sites. Colonies of the ant *T. crassispinus* discovered in nest chambers of larger volume comprised more workers than those in chambers of a smaller volume. This might be due to the fact that larger colonies prefer larger nest chambers, however, it is also possible that larger nest cavities ensure better conditions for the development of a colony, which may further affect the colony growth rate, and hence the larger number of workers. Nests were left out in the field for 1 year and collected afterwards. I have no data how long colonies have been inhabiting the nests, and the period could affect colony growth and number of workers: as the nests with the largest number of workers could just have been inhabited earlier. What more, the results could be explained by intraspecific competition also: larger colonies can be able to remove smaller colonies from larger nests. However, in such cases it means the larger nests were preferred by the ants: such nests were inhabited earlier, or there is strong competition for larger nests.

An additional problem that occurs, while analyzing the field data concerning the number of workers in differently sized nests, is polydomy—simultaneous use of several nest sites by one colony (Strätz and Heinze, 2004; Debout et al., 2007). The nest cavity size and the density of nests may impact on polydomy (cf. Debout et al., 2007; Cao, 2013). In the study by Bialas et al. (2011) conducted in the same area, an average of 2.5 nests/m^2^ in June and 1.6 nest/m^2^ in August were found. The number of nest sites available in the above-described field experiment was large (four artificial nests added per square meter) and their density could have affected polydomy, as I discovered queenless colonies in approximately half of the cavities (Fig. 2), which might have resulted from the simultaneous use of more than one nest site by a colony. Genetic data can be used in the future to determine which nests belonged to single colonies.

Nest cavity size is undoubtedly important for colonies with large numbers of individuals. In late summer and autumn, *T. crassispinus* ant colonies may consist of over 300 workers (Mitrus, 2013; and unpubl. data). Considering that the colonies containing ~150 workers could not fit into 470 mm^3^ nests during the laboratory experiment, and that *Temnothorax* ants are subject to intense competition for nest sites (Herbers, 1989; Herbers and Banschbach, 1995; Foitzik and Heinze, 1998), the choice of a bigger nest may ensue from the perspective of colony growth in the season, since the ants of this species make only minor modifications to the nest sites they find.

It was shown in previous research that colonies living in inferior nest sites invest more energy in males than females, producing a more male-biased sex allocation ratio (Strätz and Heinze, 2004). In the combined field and laboratory experiment queenright colonies inhabiting larger nest chambers (and at the same time containing more workers) invested more energy in sexuals, but no relationship between the chamber size and the proportion of males among sexual individuals was found. However, in the studies, to estimate the cost incurred of producing sexual individuals, I adopted literature data for the ant *T. nylanderi* (Foitzik and Heinze, 2000; Foitzik et al., 2003), as no such data for *T. crassispinus* are available. In the experiment sexual individuals found outside the nest chamber were removed from the Petri dishes. Such sexuals, which left the chambers, could be found dead in Petri dishes several days later. But sometimes only parts of their bodies remained in the dishes (pers. obs. from previous experiments). Thus, it could be difficult to count them accurately; therefore I decided to remove such sexuals from the dishes. However, I think that removing the individuals not affected production of other sexuals, and on sex ratio, as I removed only the ones which oneself left the nest chambers, and I started to remove them on 24 July (experiment ended on 6 August).

In ants per-capita productivity often declines in larger colonies. It is true also for six out of the eight cavity-nesting species analysed by Kramer et al. (2013). However, *T. crassispinus* did not show a decrease in per-capita productivity with increasing colony size (Kramer et al., 2013). I analyzed production of sexual individuals only, but generally my results are in accordance with the data (cf. Kramer et al., 2013). In the laboratory experiment I did not observe any correlation between the investment in sexuals and the proportions of both sexes in the laboratory experiment, as shown, e.g. in the study by Strätz and Heinze (2004).

Most ants build their nests on their own and can develop/enlarge them if need be. The nest volume can therefore be adjusted to the needs of a growing colony. Cavity-nest ants of different species can use nest chambers of various volume (Herbers and Banschbach, 1995). Previous studies have demonstrated that ants of the genus *Temnothorax* show considerable selectivity with respect to nest sites (e.g. prefer darkened sites to non-darkened, as well as sites with smaller entrances), and the competition for nest sites among colonies is strong. The results of this study show the selectivity of *T. crassispinus* ants regarding the size of nest cavity—ants of the studied species prefer nests of larger volume—and the nest volume has an impact on life history parameters, including energy allocation in sex individuals.
